# A Method of Short Text Representation Based on the Feature Probability Embedded Vector

**DOI:** 10.3390/s19173728

**Published:** 2019-08-28

**Authors:** Wanting Zhou, Hanbin Wang, Hongguang Sun, Tieli Sun

**Affiliations:** 1 School of Information Science and Technology, Northeast Normal University, Changchun 130117, China; 2 Department of General Computer, College of Humanities and Science of Northeast Normal University, Changchun 130117, China

**Keywords:** word embedding, latent Dirichlet allocation, feature weighting, text representation

## Abstract

Text representation is one of the key tasks in the field of natural language processing (NLP). Traditional feature extraction and weighting methods often use the bag-of-words (BoW) model, which may lead to a lack of semantic information as well as the problems of high dimensionality and high sparsity. At present, to solve these problems, a popular idea is to utilize deep learning methods. In this paper, feature weighting, word embedding, and topic models are combined to propose an unsupervised text representation method named the feature, probability, and word embedding method. The main idea is to use the word embedding technology Word2Vec to obtain the word vector, and then combine this with the feature weighted TF-*IDF* and the topic model LDA. Compared with traditional feature engineering, the proposed method not only increases the expressive ability of the vector space model, but also reduces the dimensions of the document vector. Besides this, it can be used to solve the problems of the insufficient information, high dimensions, and high sparsity of BoW. We use the proposed method for the task of text categorization and verify the validity of the method.

## 1. Introduction

Massive amounts of data are created on the Internet every day. It is clearly the best choice to process these data with computers. Transforming these texts written with natural languages into the forms that computers can understand has been one of the core goals of NLP. It is time consuming and labor intensive to label data sets, which generally requires a great deal of much more manual work. Therefore, unsupervised text representation is becoming becomes more and more practical. Many tasks need it, such as text classification and sentiment analysis [1,2,3].

The simplest text representation is the model is bag-of-words (BoW) model. It regards each word as a separate individual. It represents a document d as $d=(w_{1},w_{2},\ldots ,w_{l})$, where $w_{i}$ represents the ith word appearing in document *d*, and *l* represents the number of words in document d. If a corpus C contains *m* documents, the vocabulary V of C is the length of the document vector. Obviously, when the corpus is relatively large, the BoW model will have the problem of high dimensional sparsity. Since the BoW model does not consider the relationship between words, semantic information such as word meanings and context association are also ignored [4].

Taking as an example a sentence s, “I have an Apple phone”—suppose BoW incorporates the terms {have, apple, phone} into the calculation, then the vector expression of s is s=[0,1,0,1,1]. In this model, words are simply expressed with single values. Many classic feature weighting methods of BoW are statistics of words in different ranges of the corpus, and then these statistics are used to calculate the weight of words to construct the document vectors. Due to weight calculations, the final document matrix is no longer monotonously constructed by 0 and 1. These statistics may include the number of occurrences of a word in a single text, in all texts, or the number of occurrences in different categories of the corpus.

It is beneficial to use more abundant values than 0 and 1 to characterize the vectors. Some classic methods can accomplish feature selection while performing feature weighting. Calculating by algorithms such as mutual information (MI) [5] or information gain (IG) [6], one can give different weights to features. Based on these weights, feature selection is possible. Feature selection can reduce the dimensions of the document vectors. After the feature selection is completed, no further weighting work is performed, and vector representations of texts can be obtained.

Feature engineering obtains shallow models. Shallow models have a bottleneck because the texts contain more semantics than the models’ hypothesis [1]. In recent years, some approaches for the research into deep learning have involved NLP. On the whole, there are five major types of models: word embedding (e.g., Word2Vec [7], FastText [8], Glove [9]), convolution neural network feature extraction [10,11], the context mechanism [12], memory storage mechanism [13], and the attention mechanism [14].

Thus, there are usually two basic kinds of models that can be used for text representation. They are shallow models such as feature engineering and the deep models based on deep learning. Concomitantly, there are three schemes that can be used for text representation:

1. Improve the shallow models: The shallow models also have a great deal of room for improvement through the idea of dimension reduction and improved weighting methods. The works [15,16,17,18,19] introduced in Section 2 have achieved good results.
2. Develop deep models: This is a major research direction, and deep learning models such as Bidirectional Encoder Representations from Transformers (BERT) [20] have achieved excellent results. Deep models can capture semantic information better [7,21]. For example, the Embeddings from Language Model (ELMo) [22] considers the used context to train word vectors. It can obtain different word vectors for one word. Different word meanings correspond to different vectors, while general word vector models, such as GloVe, can get one fixed word vector for one input word.
3. Combine the achievements of the shallow models and the deep models: Some researchers [1,23,24] have tried this route on different tasks. Although feature engineering generally does not introduce deep learning models and deep learning-based models do not need feature engineering, they can work together as long as words or documents can be expressed as vectors. The effect of these methods can also be compared by the same classification task.

Therefore, this paper considers the introduction of word embedding. The new models we propose can create document vectors for short texts. They are unsupervised, simple, easy to understand and can be used even if there is only a small data set.

Furthermore, this paper also attempts to use topic models for document modeling. Topic models are also shallow models, but they are completely different from the feature engineering. Feature engineering traditionally gives zero value to words that are not included in a text. These locations, which are originally 0, can be utilized with the help of topic models. A word in a corpus may not appear in the text, but this does not mean that the words of the text have no association or similarity to it. By clustering the words in the corpus into several clusters, the similarity between words could be discovered. Words clustered into one cluster can be regarded as a topic. Usually, a document only discusses a few topics, and a sentence in the document is also around the topic. For the sentence s mentioned above, suppose that the probability that belongs to the ‘phone’ topic is 0.9, for a collection of words {I, have, an, Apple phone}, if the probability of these words belongings to the ‘phone’ topic is {0.1, 0.1, 0.5, 0.1, 0.2}, then sentence s could be represented as s=[0.09,0.09,0.45,0.09,0.18].

If a document representation matrix is formed by sentence vectors such as s, it would not have many 0 values. However, compared with the matrix obtained by the traditional feature engineering, topic models only change the value itself but do not represent the word as a multi-dimensional vector. Therefore, this paper considers using the method of word embedding [7,25], which can address the issue of lack semantic information in BoW by conversing words into vectors. 

**Figure 1:**
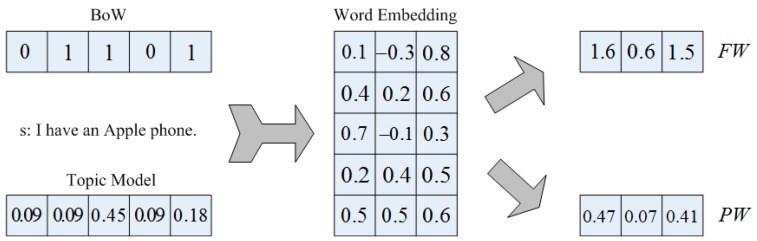
Basic idea of the feature and word embedding (FW) model and the topic probability and word embedding (PW) model. BoW: bag-of-words model.

Assuming the word embedding vector matrix shown in Figure 1 is utilized, the representation of sentence s can be obtained as FW=[1.6,0.6,1.5] or PW=[0.47,0.07,0.41]. According to the result of the operation, the original five dimensional sentence vector becomes three dimensions, but there is no loss of information either obtained from the feature weighting or topic model. Furthermore, the semantic information of the word itself is also added. As our experiments show in the next section, the proposed model that combines the traditional methods and deep learning is optimal for text representation.

In this paper, the core idea of text representation is shown in Figure 1. The models shown are the feature and word Embedding (FW) model, topic probability and word embedding (PW) model. Based on these two models, we propose the feature, probability and word embedding (FPW) model.

At the same time, this paper proposes a text representation model for splicing FW and PW, named the FW and PW conjunction (FPC) model. In addition, with reference to the work of lda2vec [26], we propose a text representation model that combines the FW model and the second kind of topic probability model (FP2). The details of all models will be described in Section 3. The work related to this paper will be described in Section 2.

## 2. Related Work

Currently, word embedding vectors have become a popular research object. The idea of the word vector was first proposed by Hinton [27] in 1986. The core idea is to make a distributed representation of a word, expressing each word as an n-dimensional dense, continuous vector. The main advantage of distributed representation is the fact that it has very powerful feature representation capabilities, such as k values of n-dimensional vectors per dimension, which can represent $k^{n}$ concepts. In fact, both the hidden layer of the neural network and the probabilistic subject model of multiple latent variables are distributed representations. In 2003, Bengio proposed the neural probabilistic language model (NNLM) [28], which was used to represent each word as a dense vector.

After that, the word vector became a popular research object until the release of Word2Vec by Google in 2013. Word2Vec proposes two models, namely continuous bag-of-words (CBoW) model and Skip-Gram, which are similar in structure to NNLM. However, they use a different vector coding idea, namely a one-hot encoding vector space. Each vector has only one dimension of 1, and the rest are 0. According to Mikolov’s work [25], the accuracy of the Skip-Gram model is superior to the CBoW model. The Skip-Gram model can achieve an accuracy of about 60% in various experiments, but CBoW can only reach about 40%. Thus, this paper adopts the Skip-Gram model, which predicts the context of a given input word. Once the model is built and trained, the weight matrix of the hidden layer of a linear neural network in the model can be obtained. This weight matrix is the word vector matrix.

The word vector obtained by Word2Vec can be used for various tasks, such as calculating the similarity of words and finding the relationship between words based on semantics. This paper uses these vectors to express texts. The main idea is to combine new technologies with traditional technologies, so it also needs to rely on traditional text representation methods.

Feature selection and feature weighting are common tasks for traditional text representation methods. This paper chooses the most basic and simple feature weighting algorithm, namely the term frequency—inverse document frequency (TF–IDF) [29], where TF refers to the word frequency and *IDF* refers to the inverse document frequency. Its equation is (1)

(1) $$tfidf_{w}=tf_{w}\times \log_{2}(\frac{n(D)}{n(D_{w})})$$

where $tf_{w}$ indicates the frequency of word w, n(D) indicates the amount of texts in set D, and $n(D_{w})$ indicates the amount of texts that contain w.

The number of statistical words appearing in texts and the corpus can represent the importance of words. The importance of a word increases proportionally with the number of times it is contained in a document, but it also decreases inversely with the frequency it appears in the corpus. As an unsupervised feature weighting method, *TF–IDF* can complete text representation even for data sets without label categories. However, as noted in Section 1, traditional feature weighting techniques, such as TF–IDF, are inclined to represent multiple texts as a large sparse matrix, which may cause high-dimensional representation [30,31,32,33]. 

Depending on the idea of feature weighting, many researchers have proposed different feature selection methods. For example, Zhou [15] proposed the interclass and intraclass relative contributions of terms (IIRCT) model, introducing the concept of intra-class and inter-class frequencies. Chen [16] proposed the term frequency and inverse gravity moment (TF–IGM) to examine the influence of features on different categories. Researchers have proposed some feature selection methods for specific tasks. For instance, Parlar’s query expansion ranking (QER) method [17] is suitable for sentiment analysis. Zheng [18] introduced lexical and syntactic information to supplement the selection of features. In addition, it is also feasible to optimize existing feature selection methods [19]. 

These new methods have achieved satisfactory results. As far as the core idea is concerned, all the above methods use feature weighting to perform feature selection. We also perform feature engineering, but we want to reduce the dimensionality of the eigenvectors. Moreover, many of these new feature-based engineering methods incorporate category information, which is supervised learning because of the need to count the frequency of features appearing in different categories. The goal of this paper is to obtain unsupervised methods, so we do not add category information. In order to make the models easy to understand, we have not attempted to introduce lexical or syntactic information.

**Figure 2:**
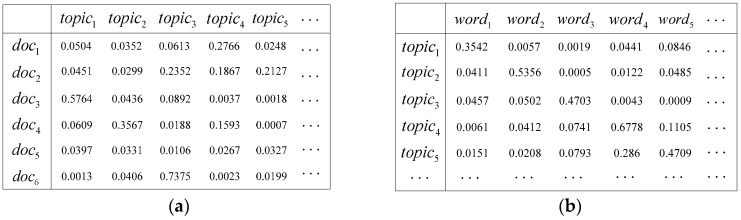
Assuming that there are 6 documents (texts) in a corpus, the (**a**) document—topic distribution and (**b**) topic—word distribution are as depicted.

Another technique that can be utilized to represent documents is the topic model. The idea of the topic model is conceptually different from feature engineering. In the perspective of the topic model, there is a hidden hierarchy named ‘topics’ from words to chapters. As latent Dirichlet allocation (LDA) [34], a classical topic model, believes, documents are usually talking about one or several topics, so the words involved will be mostly focused on these topics. As long as the appropriate algorithm is used, the words appearing in the document can be clustered to understand the topic being discussed. For example, an article about an Apple mobile phone may have several words regarding appearances, speed, screen size, clarity of the photo, overall cost performance, etc. If we put together and observe these words, we can generally determine what the article mainly says about the mobile phone. Structures of the document—topic distribution and the topic—word distribution of LDA are set out in Figure 2.

Since all of the words are involved in modeling, the dimension of the LDA topic—word matrix is the size of the vocabulary V; that is, all words in the vocabulary will have a probability value for each topic. Although LDA is usually used to cluster words, it can also be used for text representation. Numerous studies have attempted to improve LDA in order to make those words belonging to a topic have a greater probability under the topic while a lower probability under other topics. Such approaches can increase the discrimination of different topics. These studies have also achieved considerable results, but we consider the complexity of the actual operation only using the basic LDA model.

In this paper, the reason to use LDA for document modeling lies in the essence of LDA topic modeling. LDA concerns how an article is written from a topical point of view, unlike feature engineering. Although it is usually only used for topical clustering, it has a different perspective. This kind of modeling can obtain some information that traditional feature engineering does not care about. Traditional feature engineering generally does not care about words that are not included in a specific text. However, in the LDA topic model, even if a word w is not mentioned in an document d, as long as d discusses topics related to w, it can be considered that d has a certain relationship with w. Therefore, after the concept of a ‘topic’ is introduced, documents will be represented as probability vectors of topics. Through the probability vectors of topics and words, the degree of association between documents and words can be obtained.

As mentioned above, we also refer to the idea of an advanced work lda2vec [26] of LDA. Lda2vec is a model that combines LDA and Word2Vec to construct context vectors. It considers that the document vectors are added to the word vectors to obtain the context vectors. In LDA, if we want to visualize what an article is discussing, we are required to sort the probability values of the words in each topic, find the words with higher probability and print them out. Lda2vec uses an algorithm similar to LDA, but unlike LDA, the topic–word matrix is truncated after being sorted. The resulting document vector dimension is only comparable with the word vectors by Word2Vec, such as 300 dimensions [35]. This is intended to facilitate the calculation of the context vectors.

This is an inspiring idea of dimensionality reduction: retaining more important information and deleting less-worthy parts, then matching with the results of other methods, finally obtaining a better model. Text categorization has made many advances in the era of deep learning, but it still requires model selection, parameter optimization, coding, and preprocessing (one-hot, n-gram), as well as time factors. Therefore, it is necessary to find a simple and effective model with fewer parameters to be easily adjusted.

In addition to the above basic methods, some researchers have proposed some methods which combine deep and shallow models. For example, Zhao [1] proposed a fuzzy bag-of-words model. With the help of a simple TF—feature weighting algorithm and Word2Vec, a good text representation method is obtained. Another example is Lan’s method [36], which combines traditional similarity calculations with word embedding. It uses WordNet for word similarity calculation. WordNet is a knowledge base. Feature weighting and word vector combinations can also accomplish some key words extraction tasks, such as in the work of Chen [23] and Hu [24]. Methods of combining topic models with word vectors have also been studied. For example, WE-LDA [37] is used for word clustering. The lda2vec mentioned above considers the context vectors to improve topic extraction. The G-LDA [38] model combines Gaussian LDA with word embedding and is also used for topic extraction. This paper deems that the combined models can be used for a variety of purposes, and so we try to create some for text representation as well.

## 3. Models

This section introduces our proposed models. They all depend on word embedding, so we start with the word embedding vector.

According to Google Word2Vec, words can be presented as multidimensional vectors. Based on the training data, a neural network is constructed, and a word vector model is trained to obtain the weight matrix of the hidden layer of the neural network. These weight values form the word vector. The dimensions of the vector can be set to a fixed value, and Google sets this value to 300 in some experiments, which mean that each word is represented by 300 features. 

The word vector $w_{i}$ of one word $w_{i}$ can be expressed as in Equation (2)

(2) $$w_{i}=\left[vec_{1}{}_{|w_{i}},vec_{2}{}_{|w_{i}},\ldots ,vec_{n|w_{i}}\right]$$

where $vec_{t|w_{i}}$ is the value of the tth dimension of the word vector representing word $w_{i}$, and n is the dimension size of the vector. If we set a 300-dimensional word vector, the value of n is 300. This paper uses bold forms to represent matrices or vectors.

There is a total of v different words in the corpus C as training data to form the word list V. Every word in the word list V is encoded. Assuming that the word vector of each word is used as a row vector, and there are a total of v words in the vocabulary, a word vector matrix W with v rows can be formed. This word vector matrix W of the corpus C is described as in Equation (3)

(3) $$W=\left[\begin{array}{c} w_{1}\\ w_{2}\\ \ldots \\ w_{v} \end{array}\right]$$

In the experiment, the word vector is trained according to the default parameters recommended by Google, words with a frequency less than 5 in the entire corpus are filtered out, and the word vector is not calculated. Therefore, if we need these vectors of words with a lower frequency in the experiment, the system should randomly generate an n-dimensional vector to participate in the operation. We do not choose to change the recommended parameters because we believe this helps to judge the versatility of our approach.

### 3.1. The FW Text Representation Model

In this paper, the feature weighting technique is first used to represent the text with the word vector. The feature weighting part adopts the *TF–IDF* method as mentioned above. Supposing the corpus C consists of m documents as a text set, based on the word vector and the feature weighted text representation model, the vector $fw_{i}$ of the document $d_{i}$ is obtained by Equation (4)

(4) $$fw_{i}=tfidf_{i}•W$$

where W, as mentioned above, is a word vector matrix obtained by Word2Vec, usually fixed in hundreds of dimensions. $tfidf_{i}$ is the *TF–IDF* representation of document $d_{i}$, which can be formed as in Equation (5)

(5) $$tfidf_{i}=[tfidf_{w_{1i}},tfidf_{w_{2i}},\ldots ,tfidf_{w_{vi}}]$$

where $tfidf_{w_{i}}$ can be calculated as (1). The strategy of *TF–IDF* is to observe the entire vocabulary V and text set D, calculate the word frequency (TF) and the inverse document frequency (IDF) of words in D. The words that do not appear in the document $d_{i}$ are represented as 0 in the $tfidf_{i}$ vector. Therefore, the dimension of this vector is v, the length of the vocabulary V. This may have many zero values. Since the entire text set D has m documents, we will obtain m vectors to form a *TF–IDF* vector matrix.

After the calculation by (5), the dimension of the document vector $fw_{i}$ is equal to the dimension of the word vector matrix W. This approach can solve the dimensional problem of high-dimensional sparse matrices well. There is no requirement to perform complex clustering operations or special dimensionality reduction operations. As long as the matrix obtained by feature weighting is multiplied by the word vector matrix calculated by Word2Vec, the traditional feature weighting technique can be combined with new word embedding. The vector dimension of each text can be low, in the range of tens to hundreds, but in theory it does not sacrifice information obtained from any of the feature weighting or word vectors. Then, the text matrix FW of the entire text set D can be expressed by Equation (6)

(6) $$FW=\left[\begin{array}{c} fw_{1}\\ fw_{2}\\ \ldots \\ fw_{l}^{} \end{array}\right]$$

In experiments, in order to use it with the subsequent models, the *TF–IDF* vector matrix is normalized, as in Equation (7)

(7) $$fw_{inew}=\frac{fw_{i}-fw_{\min}}{fw_{\max}-fw_{\min}}$$

where $fw_{inew}$ represents each value in the normalized FW matrix, $fw_{i}$ represents each value in the original FW matrix, $fw_{\min}$ represents the global minimum of the FW matrix, and $fw_{\max}$ represents the maximum value in the FW matrix.

### 3.2. The PW Text Representation Model

The core idea of the topic model is to assume that a document will focus on one or several fixed topics, and we can discover these topics by aggregating words in the document. A number of documents in a field can also be summarized as such. There are a variety of topic models which can be used for topic discovery tasks.

The LDA we used is a well-known topic model and has been widely accepted by academics and industry. Using the LDA algorithm and performing Gibbs sampling [39], the topic distribution θ of documents and the word distribution φ of topics can be obtained with Equation (8)

(8) $$\theta =\frac{n_{m,k_{i}}^{\neg i}+\alpha }{\sum_{i=1}^{K}n_{m,i}^{\neg i}+K\alpha }\;\varphi =\frac{n_{k,w_{i}}^{\neg i}+\beta }{\sum_{i=1}^{V}n_{k,i}^{\neg i}+V\beta }$$

where i indicates the sequence number, m denotes the document label, k denotes the topic label, w denotes the label of the word, K is the total number of topics, V is the total number of words, α and β are hyper parameters. In experiments in this paper, the topic number K is set to 20. According to the convention, α=1.0, β=0.1, and the number of iterations is set as 1000.

Both θ and φ create a probability matrix. From θ, we can see weights of different topics in each document. From φ, we can see the weights of different words under each topic. If a document focuses on a few topics, the value of the corresponding position is larger in the θ matrix. Although LDA is also based on BoW, it does not consider the connection between semantics and neighboring words, but it can find words that often appear under a certain topic. In view of this, if we can determine the core topic of a document, even if some words that appear frequently belong to the topic do not appear in the document, we can believe that this document is relevant to these words.

After obtaining the relation matrix of the document and words, we can get the document vector by using Word2Vec. According to this idea, we can obtain a simple text representation model **PW**, such as in Equation (9)

(9) PW=θ•φ•W

In the lda2vec model, some changes are made to the φ matrix. This paper refers to its idea and also gets a document representation model without using Word2Vec. The φ matrix expresses the probability of a word under each topic, which can be seen as the weight of each word belonging to a topic. In any topic, a large probability value indicates that the corresponding word is more representative of the topic. LDA takes all the words into consideration. However, in fact, each topic will only have some representative words. Therefore, we consider extracting these words to form a new topic—word matrix. Therefore, we can reduce the dimension of the φ matrix. 

We traverse the φ matrix by row, sort from large to small, and put the words with the highest probability under each topic in the front row, intercept the vector of fixed dimension, and obtain the new matrix $\varphi {}^{\prime }$. That is a new text representation model P2, as shown in Equation (10)

(10) $$P2=\theta •\varphi {}^{\prime }$$

In experiments, the dimension of $\varphi {}^{\prime }$ will be set to the same as the word vector from Word2Vec to facilitate a comparison with other models.

### 3.3. The FP2, FPW, and FPC Models

By combining word vectors with feature weighting and topic models, we obtain two different models of text representation. Now, we let the two models cooperate with each other to obtain the final text representation models. The combination is expected to outperform each model separately.

Currently available text representation models, combined with vectors of two different strategies, are commonly in conjunction or extending. For example, some similar words of a word are added to the document matrix as extended dimensions. If the **FW** model is added to the document vector calculated by the **P2** model, a document vector representation model **FP2** can be obtained, as shown in (11)

(11) FP2=FW+P2

We note that Word2Vec and lda2vec all mention one opinion about word vectors; that is, the addition of word vectors can also represent semantic combinations. Based on this, we propose a new document vector combination model **FPW**, as shown in Equation (12)

(12) FPW=FW+PW

The document vectors obtained by **FW** and **PW** are summed to obtain a set of document vectors that can cover the two models. In addition, this paper also tries a conjunction model **FPC**, as shown in Equation (13)

(13) FPC=[FW,PW]

**Figure 3:**
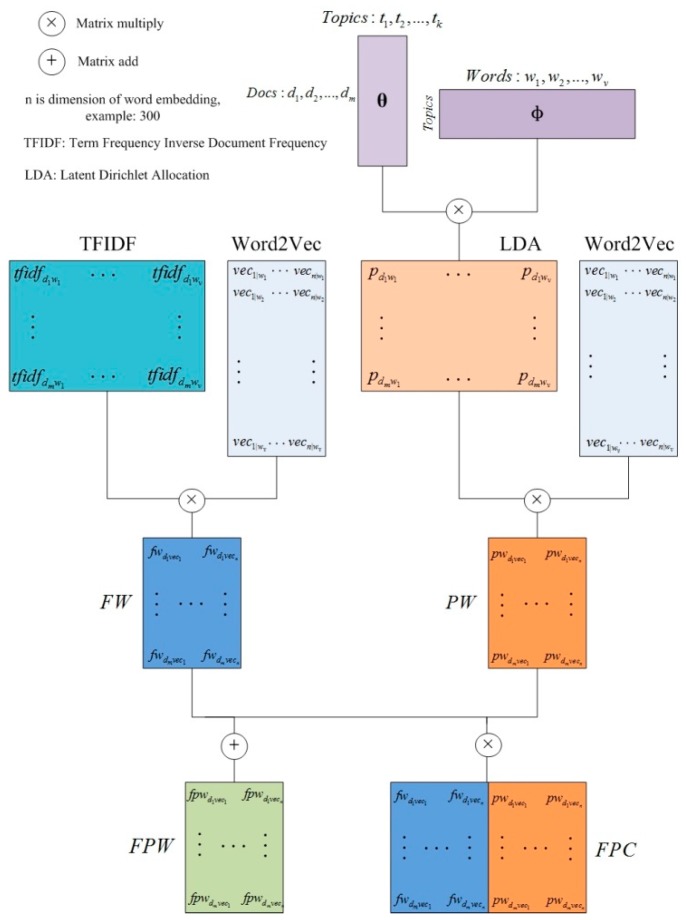
Feature, probability, and word embedding (FPW); and feature and word embedding (FW) and topic probability and word embedding (PW) conjunction (FPC) models.

**Figure 4:**
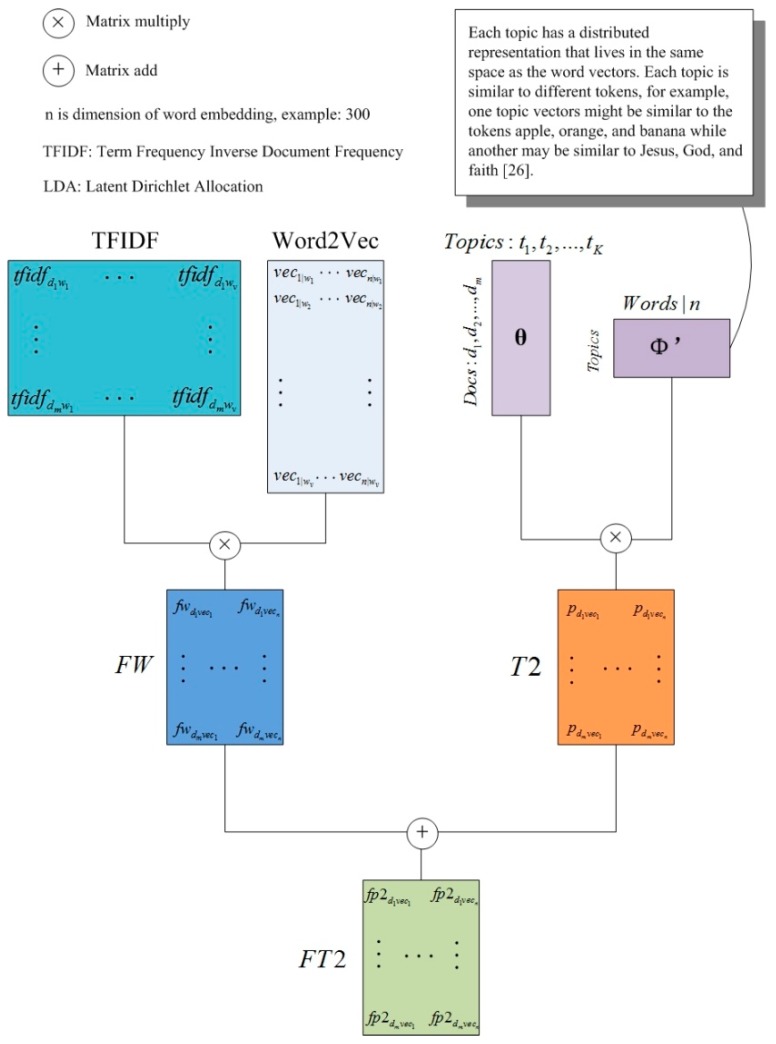
Second kind of feature probability (FP2) model.

The overall ideas of the above models are demonstrated in Figure 3 and Figure 4.

## 4. Experiments and Discussion

In this part, we use the common algorithms of text categorization to evaluate our models. The text representation models we proposed are unsupervised, but in order to prove their validity, experiments are carried out using already labeled data sets.

With regard to the task of text classification, some current research is carried out on very large data sets and involves extreme multi-label text classification [40,41,42,43]. When an article may have multiple category labels, it is necessary to conduct specialize research in classification algorithms. The portion of the text representation can still utilize the *TF–IDF* algorithm, but the category labels may also require a vector representation. It may also be necessary to train a linear classifier for each label. In order to focus on the text representation method, this paper adopts the experiment of single label classification.

### 4.1. Corpus

There are various corpora of text classification. This paper selects corpora of public data sets in Chinese and English. They are also used to train word vectors. These data sets are not too large in scale, but they all have practical value. In realistic applications, although people can obtain a large amount of text data without limiting their scope, it may be necessary to collect data for a specific topic within a certain period of time. For example, if reviews are collected for a definite product of a brand, the amount of data obtained in a single category may be small. Therefore, it is still necessary to test the method of this paper on general-scale data sets.

Amazon_6 [44] is a corpus for sentiment analysis tasks, which are used for classification. This corpus has six categories, namely cameras, laptops, mobile phones, tablets, TV, and video surveillance. There are differences in the corpus size under different classes in the original corpus. This problem still exists after preprocessing. For example, the tablet category has only 896 texts, while the camera category has 6819 texts. We select the camera and mobile phone categories to experiment. In order to keep balance, we sort the shorter 4000 texts according to length. Then there are 2000 texts in each category, and a total of 17,565 different words involved in the operation. The text data of the other four classes are lower in number than the camera and mobile phone. In order to test the effect of our method on the unbalanced but real dataset, we experiment with short texts in these four classes. Considering that the data is stored in ‘txt’ files, the short texts of this experiment refer to texts that are shorter than 50 KB in size. The total number of texts participating in the experiment is 5705, and the vocabulary size is 48,926.

FudanNLP [45] is an available corpus for the Chinese text classification task disclosed by Fudan University, China. It contains 20 categories. The number of texts in each category is different. In order to maintain balance, this paper chooses three categories: computer, economy and sports. Five hundred texts are selected from each category for experiment. In addition, similar to the Amazon_6 corpus, we use the remaining 17 classes of data to do the unbalanced experiment. In the case of a comparable amount of information, Chinese articles usually take up less space than English, so a small portion of the texts in the data set with a capacity greater than 15 KB were not included in the experiment. A total of 4117 texts were included in the experiment, and the vocabulary size was 78,634. The difference in the number of texts in the 17 categories is large. For example, there are only 59 texts in the transport class but 617 texts in the space class. However, due to the large number of classes, the vocabulary is still large.

ChnSentiCorp [46] is a sentiment analysis corpus released by the Beijing Institute of Technology. It contains data in the fields of laptops and books. Each field contains 4000 short texts, which are marked with positive and negative emotions. In each class, the number of texts is 2000. All the data are used for experiments. The vocabulary of laptops is 8257 and the class of books is 22,230.

In summary, this paper selects two kinds of Chinese and English data sets, which are the corpora of general classification and sentiment classification. The corpora selected by the sentiment classification task are different from general texts, and usually contain subjective emotional expressions of human beings, while there is less content of the general character. In this way of labeling, the corpus for sentiment analysis may also different from the general corpus. It is possible to label positive and negative emotional polarities for each field, which is unnecessary for general text data sets. Thus, this paper selected these different data sets for the experiments to prove the applicability of the models.

### 4.2. Preprocess

For the English corpus, we use the NLTK library with Python for preprocessing. The preprocessing includes the usual removal of punctuation and stop word processing, as well as root restoration. The English stop words list comes from NLTK. Raw Amazon_6 texts are json files, so we extracted the content parts of the json files. These parts are comments of commodities. Then we made txt files for experiments. One json file corresponds to one txt file. 

For the Chinese corpora, we use a java version of the word segmentation tool developed by the Chinese Academy of Sciences ICTCLAS [47] to segment words, and then remove stop words and punctuation. The Chinese stop words list comes from CSDN [48]. It contains 1208 stop words. Before the text representation calculation, it is considered that ordinary words in Chinese include generally less than five Chinese characters, so words with a length greater than five are removed.

Regarding the setting of word embedding, Word2Vec can set the dimension of word vectors. We set dimensions of 200, 300, 500, 800, etc., to compare the effects of the models in different vector dimensions. Three hundred is a common setting for Word2Vec papers [7,35]. Our specific test values are 200, 500 and 800, with increments of 300.

In addition to the change in dimensions, other parameters are always maintained in the conventional settings of the Word2Vec tool. Since Word2Vec does not calculate word vectors for those words used less than 5 times, we randomly generate vectors with values between (0, 1) for these words.

In this paper, the *k*-nearest neighbor algorithm (KNN) classifier [49] and linear support vector machine (SVM) classifier [50] were used to classify texts and perform a 5-fold cross-validation with an average accuracy rate. SVM is a well-recognized classifier. KNN has very good classification effects on the LDA document models, but it may behave generically with the feature weighting models. By observing the performance of two different classifiers, we can better evaluate our models. We adopted the Sklearn tool to randomly set the k value for testing. In Linear SVM, we set the parameter C as 1.

### 4.3. Measure of Performance

This paper uses the accuracy and F1-score [51] to measure the effect of classification. Accuracy is a common metric in deep learning research, and F1 is a common criterion for classification tasks. When it comes to multi-classification, this paper uses F1-macro. 

Suppose there are two classes labeled positive and negative: *TP* indicates the number of documents correctly classified into the positive class, *TN* indicates the number of documents correctly classified into the negative class, *FP* indicates the number of documents classified into the positive class incorrectly, and *FN* indicates those that are assigned to the negative class incorrectly. Then the algorithm of accuracy is shown in Equation (14)

(14) $$Accuracy=\frac{TP+TN}{TP+TN+FP+FN}$$

Among the two classification problems, there are two evaluation criteria of precision and recall [52], as shown in Equations (15) and (16)

(15) $$P=\frac{TP}{TP+FP}$$

(16) $$R=\frac{TP}{TP+FN}$$

The F1-score can take these two criteria into account

(17) $$F1\_score=\frac{2*P*R}{P+R}$$

In the multi-class problem, we can first count precision and recall for each class, and then calculate the arithmetic average to obtain the *F*1-macro. This is a recognized and convincing evaluation criteria:

(18) $$Macro\_P=\frac{1}{n}\sum_{C=1}^{n}P_{C}\;Macro\_R=\frac{1}{n}\sum_{C=1}^{n}R_{C}\;Macro\_F=\frac{1}{n}\sum_{C=1}^{n}F_{C}$$

Therefore, this paper uses both accuracy and *F*1-macro to measure classification effects.

### 4.4. Experimental Results and Discussion

This part gives the experimental results and the corresponding analysis. First, we analyze a single set of experiments performed on each corpus, and then give an overall discussion. The baseline methods are TF–IDF, LDA, and Word2Vec. Word2Vec refers to the method of simply adding the vectors of words to represent an entire document. The results of the different dimensions are averaged and presented in tables. The best results in each column are shown in bold.

Experimental results of Amazon_6 corpus are shown in Table 1 and Table 2. The results are good overall, but the models proposed in this paper add a certain accuracy rate and *F*1-value compared with the baseline. 

Table 1 shows that the FPW model maintains a relatively high accuracy, and the FPC model is close behind. Since the FPC model is a conjunction of two matrices of FW and PW, its vector dimensions are more than marked in the table, which are 400, 600, 1000, and 1600, respectively. If we consider comparing the accuracy in approximately equal dimensions, then the FPW model is still better, and it only uses hundreds of vectors to get better results.

Table 2 gives the experiments of the Amazon_6 corpus by SVM. FPW and FPC have excellent overall effects. The results of FPW are stable and the best. The global maximum value appears in the 500-dimensional experiment of FPW. The PW model has an improvement compared to the baseline LDA, but FW is slightly worse than TF–IDF, which may occur on some corpora. The classification decision function of SVM is determined by only a few support vectors, not the dimensions of vector space. After reducing the dimensions of the vector space, the resulting new vector incorporates more information, but it may also increase the difficulty of classification to some extent. However, after further integrating the vector information of PW, a better classification effect can still be obtained.

At the same time, in this set of experiments, P2 has lost a great deal of information because the part with fewer probabilities in the LDA φ matrix is cut off. The effect is not good. However, after combining with word embedding, there is obvious improvement. It can be observed that the information obtained by introducing more vectors is helpful for the vector representation of texts. This is also evident in the experiments of the SVM classifier of the following corpus.

In the following Table 3 and Table 4, this paper uses the data from the other four categories of the Amazon_6. This set of experiments involves an unbalanced data set with a few more categories.

This set of experiments performed on the KNN classifier results in the best effect on the PW model. The accuracy of FPW in the 500 and 800 dimensions exceeded the baseline methods, and FPW and FPC both exceed the baseline methods on F1-macro. Since the values of the vectors obtained by *TF–IDF* and Word2Vec are both positive and negative, it may happen that FW is slightly worse than *TF–IDF* or Word2Vec. The vectors of LDA modeling are only positive, so in most experiments, the effect of PW will be better than LDA or Word2Vec. In the next experiment, such a situation can also be observed. In addition, P2 is also effective, and the impact of data imbalance is not very large. The FP2 model is unexpected but has improved over the P2 model.

Table 4 shows that FPW performance is the best for SVM, followed by FPC. The effect of FW is much lower than the baseline methods, which may be related to the imbalance of the data. The effect of P2 is also poor. It can be seen that SVM does not adapt to the model truncated by topic modeling in this corpus. However, the full PW still performs well, exceeding all baseline methods. The results of the two classifiers above show that the method of this paper can also achieve a certain improvement effect on the unbalanced data.

In the work of Zhao [1] mentioned in Section 2, the Amazon_6 corpus is also used. In order to balance the data, Zhao randomly selected 1500 texts in 5 classes which contain more than 1500 texts, with a total vocabulary of 10,790, and experimented with the SVM classifier. In the end, the fuzzy bag-of-words model has proved very effective, with a classification accuracy of 92% to 93%, which was 1% to 2% higher than the baseline TF (BoW) and LDA. This paper does not select texts randomly and the vocabulary is slightly larger. However, we also use LDA as a baseline method, and use *TF–IDF* as a benchmark, which is better than TF. The above two sets of experiments show that the method of this paper has a similar increase, which is about 1% to 2.5%.

Table 5 and Table 6 gives the experimental results related to the FudanNLP corpus. This set of experiments is still performed on FudanNLP.

Table 5 shows that LDA is effective, while *TF–IDF* is general. Therefore, most of the better results appear in PW, which means the combination of LDA and Word2Vec gives better results. The best values for the accuracy and F1-macro of our models exceeded the baseline methods, but the best results appeared in different methods. It can be observed from subsequent experiments that such a situation occurs with FudanNLP more than once, but other data sets have no such phenomenon, so we speculate that this situation is related to the data set.

In experiments with SVM on the FudanNLP corpus, FPW and FPC still achieve better results, and the overall results are better than the baseline methods. The global maximum value appears in the 300-dimensional experimental result of FPC. Similar to the results of experiments on Amazon_6, the effect of FW decreases slightly, but PW gets better results. The P2 model is the worst, but FP2 can significantly improve accuracy. In the experiments on P2 and FP2, the benefit of introducing more information can be seen obviously.

Table 7 and Table 8 show the experimental results of the unbalanced data sets performed on the FudanNLP corpus. There are 17 categories involved and the data is unbalanced. Among the three baseline methods, except for the effect of *TF–IDF* on the KNN classifier, both LDA and Word2Vec have a significant decline from the previous experiment. However, our models still show improvement.

Among the results of the KNN classifier, FPW and FPC are the best, and most of the results exceed the baseline methods. Both FW and PW failed to exceed the baseline methods, but their combination has made significant progress. Both P2 and FP2 have average effects, but are still acceptable in terms of accuracy. The F1-macro is general and may be caused by too many categories.

In experiments conducted by SVM, FPW, and FPC exceed the baseline methods most of the time. Word2Vec itself has achieved good results. *TF–IDF* also performs well on F1-macro. The effect of P2 is still general. SVM is still unsuitable for such incomplete topic models. In the LDA method, the effect of SVM is often not as good as KNN, which is related to the classifier. The SVM classification depends on the support vectors. The text representation models based on the topic models find it relatively difficult to find the support vectors of the edges, while the KNN finds it easier to obtain the center of categories, so the vectors close to the center are divided into one class.

In the work of Zhang [53], the FudanNLP corpus also used the methods with and without normalization, and the accuracy reached 54% and 79.6%, respectively. It can be observed that this data set is extremely unbalanced, so it is not easy to get better classification results. The effects of the models of this paper are acceptable. Regarding accuracy as a standard, the models of this paper could reach about 83%.

Table 9 and Table 10 begin with the experimental results of the laptops domain of the ChnSentiCorp corpus. Since this dataset is labeled with positive and negative emotional polarities for each field, we have experimented in each field.

The corpus of laptops has 4000 texts and a vocabulary of 7892. In this set of experiments, the performance of each model is stable. FPW is the best, FPC is second, and PW is third. This may be related to the data in the corpus itself. There is a gap between the *TF–IDF* method as a baseline and LDA. Similar to the experiment in which FudanNLP selected 1500 corpus in three categories, the effect of the FW model on classification shows a significant improvement.

In the results of SVM on the laptop corpus shown in Table 10, FPC’s overall effect is better. FPW achieves good results and is better than the baseline methods. In addition to the improvement of PW compared to LDA, FW also improves in most dimensions compared to TF–IDF. The FP2 model also yields mostly better results than the baseline methods. SVM is always effective for the vector space models constructed by TF–IDF. Compared with the KNN experimental results, FW is not much better than TF–IDF, but it can obtain some improvement.

Table 11 and Table 12 begin with the experimental results of ChnSentiCorp. The data in the book field in ChnSentiCorp is special. People may write some texts about the specific content of books when they evaluate books, so even if the texts are purely positive, the content may be different. 

The effect of *TF–IDF* is general, and LDA works better, but the models we proposed obtain more significant results and improve to nearly 90% accuracy. In the results of the baseline approaches, unlike the previous Amazon_6 and FudanNLP, Word2Vec obtains a slightly worse effect. In most cases, Word2Vec is often the best in the baseline methods, or the second only to the best, but this time it is only slightly better than LDA. This occasional situation does not affect the validity of Word2Vec.

In the book field of the ChnSentiCorp, it is interesting that the best experimental results of SVM in different dimensions are consistent with KNN, which are derived from FW and FP2. The global maximum comes from the 200-dimensional experiment of FW. Similar to the conclusions obtained by KNN, the dimensions of vectors do not have to be set as too large. Although neither FPW nor FPC achieve the highest value, all the results are better than the baseline. 

Table 12 shows that FW yields better results than the baseline methods in all dimensions, but PW shows a lower accuracy rate in most dimensions than LDA. It can be seen that feature weighting and topic models have different modeling ideas for texts. When FW can obtain good vector representations, PW may not get better results, and vice versa. Different classifiers perform much quite differently between *TF–IDF* and LDA.

In terms of the experiments in this paper, KNN can get useful classification results for the models established by LDA, but not for the vectors obtained by TF–IDF. SVM is the reverse: It always gets reliable results for *TF–IDF* and ordinary for LDA. These phenomena have a lot to do with the difference between classifiers and corpora. However, in terms of vector representations, satisfactory vector representation models can obtain satisfactory results on different classifiers, as with our FW model of the last set of experiments, as well as the FPW and FPC models proposed here. At the same time, related experiments of P2 and FP2 can also obtain similar conclusions on different classifiers.

Also using this ChnSentiCorp corpus, Zhai [54] proposed a text representation method for extracting different kinds of features. Since this is a corpus for sentiment analysis, Zhai’s approach considers extracting features that are related to sentimental tendencies. In addition, features such as substrings, substring groups, and key substrings are extracted. Finally, the highest accuracy of 91.9% can be obtained under the SVM classifier, which is a very good result [55]. In this paper, without the specific feature extraction, the accuracy is comparable or even higher. 

By comparing our models with different methods on different corpora, it can be seen that the method of this paper gives an improvement in accuracy, and the increase range is from 1% to 4%. In the design of our method, there are some differences from the existing methods. Compared with the FBoW model [1], our models introduce the LDA topic model, enabling our new models to describe the texts from the perspective of topic modeling. Compared with Zhang’s method [53], the method of this paper does not need to deal with the mix problem of Chinese and English, such as word categorization. Compared with Zhai’s method [54], our method does not set special rules for feature extraction on the data of sentiment analysis, but it matched Zhai’s method in strength. 

From the above experiments as a whole, we can also gather some additional conclusions. Firstly, the dimensions of vectors do not need to be large. The 200-and 300-dimensional settings often get the best results. Secondly, for some special corpus, it is possible that the FW or PW model can get good results, but in most cases, text representation models that combine feature extraction, word vector, and topic models are more effective. Thirdly, P2 has a relatively poor accuracy due to the deletion of some information calculated by LDA. However, after combining with the FW model, even if no longer combined with the word vector, it often gets good results.

## 5. Conclusions

This paper proposes short text representation models based on the feature probability embedding vector, including FP2, FPW, and FPC. This vector is easy to implement. Although it does not require adjustment to many parameters (basically, the default parameters can be used), its effect is stable. Even if Word2Vec does not use a large-scale corpus in training, better results can be achieved. Combining different conceptual text modeling models with deep learning word embedding can make significant advances over traditional algorithms. 

In the six sets of experiments conducted on three corpora, we also made some observations about baseline methods and classifiers. Although the accuracy of classic *TF–IDF* on KNN classifiers is limited to some extent, SVM usually classifies its models well. LDA is rarely used for document modeling, but its models are often available. KNN can also achieve good results. It is also effective to add the word vectors obtained by Word2Vec to express the documents. From most of the data, the models obtained by Word2Vec can get good results on both SVM and KNN. 

Regarding the combination of methods, the FW effect deteriorates more than that of PW, which may be due to the fact that vector multiplication involves changes in algebraic signs. These positive and negative changes will change the meaning of certain values as a whole. Although the amount of information increases, new vectors may not suitable for documents. It may be better to make improvements in symbolic changes when normalized, which will make our approach more effective. However, this paper still presents relatively original results, with the aim of helping further research. This paper shows that one of the biggest benefits of the combination of methods is stability. Different methods and classifiers may perform differently in the face of different languages and different content. If we need to process different data more stably, we can consider the combination of models.

The models of the proposed text representation method focus on one idea: The combination of vectors. Since the method is purely unsupervised and the required tools and corpora are available, such a text representation strategy is simple and easy. This paper proposes a different attempt from the existing methods of vector representation. Our models do not fundamentally change existing basic strategies, but rather they can be combined as a whole in order to preserve the information obtained by the basic modeling strategy. Therefore, the operations used in this method are mostly simple matrix addition and multiplication. Experiments show that the method in this paper can also obtain good results. 

Next, we will study some new deep learning models, and different feature weighting techniques in order to obtain better text representation models. At the same time, we hope to find more interesting cooperation modes.

## Figures and Tables

**Table 1:** Results of Amazon_6 in two classes for 4000 texts by *k*-nearest neighbor (KNN).

Model	Accuracy and *F*1 of Different Dimensions							
	200		300		500		800	
	Acc	*F*1	Acc	*F*1	Acc	*F*1	*A*cc	*F*1
FW	0.924	0.9239	0.923	0.9229	0.9125	0.9124	0.9003	0.9001
P2	0.9078	0.9075	0.9075	0.9073	0.9078	0.9075	0.9083	0.908
PW	0.9555	0.9554	0.9543	0.9542	0.9548	0.9547	0.955	0.9549
FP2	0.9335	0.9334	0.9273	0.9272	0.9208	0.9207	0.907	0.9069
FPW	**0.9593**	**0.9592**	0.9555	0.9549	**0.9585**	**0.9584**	**0.958**	**0.9579**
FPC	0.9565	0.9564	**0.957**	**0.957**	0.9557	0.9557	0.9575	0.9574
**Accuracy and *F*1 of Baselines**								
**Model**	**Acc**				***F*1**			
*TF–IDF*	0.7798				0.7683			
LDA	0.954				0.9539			
Word2Vec	0.9538				0.9537			

**Table 2:** Results of Amazon_6 in two classes for 4000 texts by support vector machine (SVM).

Model	Accuracy and *F*1 of Different Dimensions							
	200		300		500		800	
	Acc	*F*1	Acc	*F*1	Acc	*F*1	*A*cc	*F*1
FW	0.89	0.8888	0.9038	0.9030	0.9218	0.9196	0.914	0.9168
P2	0.6845	0.6831	0.6853	0.6839	0.6853	0.6838	0.6853	0.6837
PW	0.959	0.9589	0.96	0.96	0.9595	0.9594	0.9605	0.9604
FP2	0.91	0.9003	0.9105	0.9099	0.9285	0.9282	0.9218	0.9218
FPW	**0.971**	**0.971**	**0.972**	**0.9719**	**0.9743**	**0.9737**	**0.972**	**0.9737**
FPC	0.967	0.968	0.9685	0.9687	0.9705	0.9717	0.9715	0.9732
**Accuracy and *F*1 of Baselines**								
**Model**	**Acc**				***F*1**			
*TF–IDF*	0.955				0.9607			
LDA	0.9488				0.9487			
Word2Vec	0.9587				0.967			

**Table 3:** Results of Amazon_6 in four classes for 5705 texts by KNN.

Model	Accuracy and *F*1-Macro of Different Dimensions							
	200		300		500		800	
	Acc	*F*1	Acc	*F*1	Acc	*F*1	Acc	*F*1
FW	0.9063	0.8818	0.8964	0.8721	0.9051	0.8817	0.9018	0.8786
P2	0.8676	0.8504	0.8678	0.8509	0.8676	0.8509	0.8826	0.851
PW	**0.9438**	**0.9325**	**0.9442**	**0.9336**	**0.9437**	**0.9327**	**0.9449**	**0.9345**
FP2	0.9167	0.8976	0.9078	0.8823	0.9167	0.8972	0.9125	0.8918
FPW	0.9427	0.931	0.9427	0.9312	0.9435	0.9319	0.9433	0.9326
FPC	0.9407	0.9283	0.9411	0.0295	0.9395	0.9276	0.9417	0.9304
**Accuracy and *F*1-Macro of Baselines**								
**Model**	**Acc**				***F*1**			
*TF–IDF*	0.8292				0.8064			
LDA	0.9253				0.9103			
Word2Vec	0.9404				0.9243			

**Table 4:** Results of Amazon_6 in four classes for 5705 texts by SVM.

Model	Accuracy and *F*1-Macro of Different Dimensions							
	200		300		500		800	
	Acc	*F*1	Acc	*F*1	Acc	*F*1	Acc	*F*1
FW	0.8038	0.7662	0.8068	0.7733	0.8334	0.8044	0.858	0.809
P2	0.6206	0.531	0.6205	0.5309	0.609	0.5348	0.6196	0.5294
PW	0.9468	0.9343	0.9442	0.9336	0.9465	0.9346	0.9472	0.9352
FP2	0.835	0.7993	0.8329	0.7969	0.8502	0.8213	0.8448	0.8197
FPW	**0.9505**	**0.9391**	**0.9507**	**0.9394**	**0.9517**	**0.9399**	**0.9502**	**0.938**
FPC	0.9488	0.9366	0.9493	0.9375	0.9497	0.9383	0.95	0.939
**Accuracy and *F*1-Macro of Baselines**								
**Model**	**Acc**				***F*1**			
*TF–IDF*	0.94				0.9366			
LDA	0.936				0.9215			
Word2Vec	0.9349				0.919			

**Table 5:** Results of FudanNLP in three classes for 1500 texts by KNN.

Model	Accuracy and *F*1-Macro of Different Dimensions							
	200		300		500		800	
	Acc	*F*1	Acc	*F*1	Acc	*F*1	Acc	*F*1
FW	0.94	0.9383	0.9353	0.934	0.93	0.9267	0.9253	0.9221
P2	0.9127	0.9119	0.9227	0.9214	0.9147	0.9139	0.9153	0.9145
PW	0.96	**0.9598**	0.9527	0.9524	**0.9607**	**0.9605**	**0.96**	**0.96**
FP2	0.934	0.9383	0.934	0.9326	0.9307	0.9284	0.926	0.9228
FPW	0.9493	0.9489	**0.96**	0.9598	0.95	0.9496	0.9513	0.951
FPC	**0.96**	0.9482	0.9527	**0.9605**	**0.9607**	0.9516	**0.96**	0.9494
**Accuracy and *F*1-Macro of Baselines**								
**Model**	**Acc**				***F*1**			
*TF–IDF*	0.5047				0.581			
LDA	0.9586				0.9584			
Word2Vec	0.9467				0.9457			

**Table 6:** Results of FudanNLP in three classes for 1500 texts by SVM.

Model	Accuracy and *F*1-Macro of Different Dimensions							
	200		300		500		800	
	Acc	*F*1	Acc	*F*1	Acc	*F*1	Acc	*F*1
FW	0.9393	0.9368	0.9387	0.9358	0.9387	0.9465	0.9473	0.9486
P2	0.602	0.5736	0.5647	0.5188	0.604	0.5774	0.6053	0.578
PW	0.9573	0.9569	0.9587	0.9585	0.9573	0.9568	0.9567	0.9561
FP2	0.938	0.9353	0.9393	0.9359	0.9493	0.9451	0.9513	0.9486
FPW	0.964	0.9635	0.9693	0.9685	0.9653	0.9649	0.9653	0.9649
FPC	**0.9673**	**0.967**	**0.97**	**0.9699**	**0.9687**	**0.9677**	**0.968**	**0.9664**
**Accuracy and *F*1-Macro of Baselines**								
**Model**	**Acc**				***F*1**			
*TF–IDF*	0.962				0.9613			
LDA	0.9406				0.9397			
Word2Vec	0.9567				0.9564			

**Table 7:** Results of FudanNLP in 17 classes for 4117 texts by KNN.

Model	Accuracy and *F*1-Macro of Different Dimensions							
	200		300		500		800	
	Acc	*F*1	Acc	*F*1	Acc	*F*1	Acc	*F*1
FW	0.7971	0.532	0.793	0.5306	0.8022	0.5328	0.7996	0.5316
P2	0.7566	0.3719	0.7575	0.3703	0.7583	0.3717	0.7585	0.3726
PW	0.796	0.4382	0.7964	0.4486	0.7928	0.4355	0.7921	0.4329
FP2	0.7973	0.5246	0.7938	0.5213	0.8023	0.5366	0.8001	0.5343
FPW	0.8303	**0.6151**	**0.8353**	**0.6136**	0.8337	**0.6043**	**0.8366**	**0.6138**
FPC	**0.8321**	0.5761	0.8351	0.517	**0.8357**	0.5612	0.8321	0.5308
**Accuracy and *F*1-Macro of Baselines**								
**Model**	**Acc**				***F*1**			
*TF–IDF*	0.6408				0.4968			
LDA	0.7979				0.4211			
Word2Vec	0.8153				0.5749			

**Table 8:** Results of FudanNLP in 17 classes for 4117 texts by SVM.

Model	Accuracy and *F*1-Macro of Different Dimensions							
	200		300		500		800	
	Acc	*F*1	Acc	*F*1	Acc	*F*1	Acc	*F*1
FW	0.7931	0.5369	0.8037	0.5552	0.8091	0.5583	0.8156	0.5856
P2	0.5317	0.3603	0.5486	0.3662	0.5563	0.3672	0.5108	0.3524
PW	0.8012	0.4436	0.8002	0.4431	0.8011	0.4437	0.8005	0.4434
FP2	0.7931	0.5356	0.804	0.5355	0.8094	0.5707	0.8152	0.5815
FPW	0.8323	**0.6327**	0.8396	**0.6465**	0.8432	0.6334	**0.8493**	0.6494
FPC	**0.8352**	0.6081	**0.8425**	0.6230	**0.8461**	**0.6376**	0.8491	**0.6629**
**Accuracy and *F*1-Macro of Baselines**								
**Model**	**Acc**				***F*1**			
*TF–IDF*	0.7628				0.6325			
LDA	0.7346				0.432			
Word2Vec	0.828				0.606			

**Table 9:** Results of laptops in ChnSentiCorp by KNN.

Model	Accuracy and *F*1 of Different Dimension							
	200		300		500		800	
	Acc	*F*1	Acc	*F*1	Acc	*F*1	Acc	*F*1
FW	0.8083	0.8079	0.813	0.8104	0.7833	0.7829	0.7633	0.7632
P2	0.7605	0.7602	0.7685	0.768	0.7623	0.762	0.7623	0.7619
PW	0.816	0.8157	0.8153	0.8147	0.8108	0.8105	0.8148	0.8144
FP2	0.81	0.8096	0.811	0.8083	0.7868	0.7864	0.7675	0.7675
FPW	**0.8383**	**0.8378**	**0.8373**	**0.8358**	**0.823**	**0.8222**	**0.8185**	**0.8178**
FPC	0.8288	0.8284	0.8343	0.8326	0.8108	0.8102	0.8095	0.8092
**Accuracy and *F*1 of Baselines**								
**Model**	**Acc**				***F*1**			
*TF–IDF*	0.5973				0.5391			
LDA	0.8065				0.8059			
Word2Vec	0.8178				0.8173			

**Table 10:** Results of laptops in ChnSentiCorp by SVM.

Model	Accuracy and *F*1 of Different Dimension							
	200		300		500		800	
	Acc	*F*1	Acc	*F*1	Acc	*F*1	Acc	*F*1
FW	0.8575	0.8574	0.8805	0.8804	0.8548	0.8541	0.8598	0.8637
P2	0.6413	0.6405	0.6187	0.6184	0.6415	0.6407	0.6415	0.6335
PW	0.8305	0.8303	0.8425	0.8422	0.8288	0.8286	0.8308	0.8306
FP2	0.8562	0.8559	0.8825	0.8824	0.8545	0.8574	0.8608	0.8603
FPW	0.869	0.8694	0.8878	0.8886	**0.8693**	**0.8687**	0.871	0.8694
FPC	**0.8703**	**0.8701**	**0.8895**	**0.8894**	0.868	0.8679	**0.8738**	**0.8712**
**Accuracy and *F*1 of Baselines**								
**Model**	**Acc**				***F*1**			
*TF–IDF*	0.8555				0.8552			
LDA	0.8195				0.8185			
Word2Vec	0.822				0.8235			

**Table 11:** Results of books in ChnSentiCorp by KNN.

Model	Accuracy F1 of Different Dimension							
	200		300		500		800	
	Acc	*F*1	Acc	*F*1	Acc	*F*1	Acc	*F*1
FW	**0.9028**	**0.9026**	**0.8988**	**0.8987**	0.8975	0.8974	0.9	0.8999
P2	0.7953	0.7932	0.77	0.7679	0.7945	0.7925	0.794	0.792
PW	0.8125	0.811	0.8208	0.8195	0.811	0.8097	0.8088	0.8074
FP2	0.902	0.9019	0.8975	0.8975	**0.8983**	**0.8982**	**0.901**	**0.9009**
FPW	0.8975	0.8974	0.8925	0.8924	0.8878	0.8877	0.8855	0.8853
FPC	0.8958	0.8957	0.8938	0.8937	0.8928	0.8927	0.8928	0.8927
**Accuracy and *F*1 of Baselines**								
**Model**	**Acc**				***F*1**			
*TF–IDF*	0.5008				0.5004			
LDA	0.7833				0.7778			
Word2Vec	0.8825				0.8823			

**Table 12:** Results of books in ChnSentiCorp by SVM.

Model	Accuracy and *F*1 of Different Dimension							
	200		300		500		800	
	Acc	*F*1	Acc	*F*1	Acc	*F*1	Acc	*F*1
FW	**0.9203**	**0.9202**	**0.917**	**0.9169**	0.9175	0.9174	0.9163	**0.9162**
P2	0.621	0.6204	0.5918	0.5912	0.6213	0.6204	0.6213	0.6204
PW	0.7783	0.778	0.785	0.7845	0.7778	0.7775	0.7778	0.7775
FP2	0.9195	0.9194	0.9168	0.9167	**0.918**	**0.9179**	**0.9165**	**0.9162**
FPW	0.9115	0.9114	0.9095	0.9094	0.9113	0.9112	0.9143	0.9145
FPC	0.91	0.9099	0.9153	0.9152	0.912	0.9117	0.9153	0.9152
**Accuracy and *F*1 of Baselines**								
**Model**	**Acc**				***F*1**			
*TF–IDF*	0.9058				0.9056			
LDA	0.7815				0.7812			
Word2Vec	0.89				0.8123			
